# Effectiveness of Sampling Techniques in Collecting the Polyp Stage of the Invasive Freshwater Hydrozoan *Craspedacusta sowerbii*

**DOI:** 10.3390/biology13080645

**Published:** 2024-08-22

**Authors:** Jonathan A. Zhu, Nadine C. Folino-Rorem

**Affiliations:** Department of Biological and Health Sciences, Wheaton College, Wheaton, IL 60187, USA; jazhu@andrew.cmu.edu

**Keywords:** Cnidaria, invasive hydrozoan, sampling, polyp, frustule

## Abstract

**Simple Summary:**

The invasive freshwater jellyfish *Craspedacusta sowerbii* exists as a microscopic polyp stage and a more visible, pelagic jellyfish-like medusa stage. Current methods to collect these polyps are challenging and time consuming, so we developed a more efficient method involving glass and plastic microscope slides. Polyps became established on these slides, confirming the efficacy of our method.

**Abstract:**

Current sampling methods for detecting the presence of the invasive freshwater hydrozoan *Craspedacusta sowerbii* rely mainly on visual confirmation of the medusa stage. Confirming the presence of the polyp stage is equally important for observing medusae since typical late summer/early fall occurrences or observations of medusae are sporadic though are becoming more frequent. The polyp stage is important as it is the organism’s primary stage and is present throughout the year depending on water temperatures. Therefore, sampling methods for the polyp stage are, commonly, the collection of substrates such as rocks, plants, or pieces of wood in a given body of water, and these can be cumbersome to examine. Polyps are also small, transparent, and difficult to see on natural substrates. Based on a preliminary culturing of the polyp stage on glass and plastic microscope slides in the laboratory, we designed a sampling methodology based on submerging four substrate types (glass and plastic microscope slides, Hester-Dendy discs, and small glass Petri dishes) to confirm the presence of *C. sowerbii* polyps in the field. We tested this method in three lakes in the Illinois–Indiana region (USA). Two of the lakes have recorded sightings of medusae but the third has no record of polyps or medusae. The sampling method we designed was effective in that *C. sowerbii* polyps were found on both plastic and glass slides. While this method can be sufficient for detection of the polyp stage, it also shows potential for improvement; we highlight abiotic and biotic ecological parameters as significant factors influencing the collection of *C. sowerbii* polyps to be considered for future methodologies.

## 1. Introduction

Invasive species have become more prevalent across the globe due to rising temperatures and human activity; these factors increase the available range of invasive species and allow them to reach more places [1,2,3]. While total elimination of an invasive species is often impossible, the most successful efforts to curb the spread of certain invasive species have been successful partly due to increased awareness and knowledge of the species in question [4,5,6,7]. Since the spread of invasive species poses a major threat to global biodiversity, it is critical to understand the impact of invasive species to develop control protocols as needed [8,9,10].

Aquatic ecosystems are especially vulnerable to invasive species given increases in temperatures and competition with native species [11,12,13]. More specifically, invasive cnidarians have already inflicted recorded damage to marine ecosystems [14,15,16,17]. However, much less work has been conducted on freshwater cnidarians, despite the fact that they may pose just as much of a threat as their marine counterparts [18,19]. While many species of invasive cnidarians exist in freshwater and marine environments, we specifically focused on the hydrozoan *Craspedacusta sowerbii* Lankester, 1880 (Cnidaria, Hydrozoa, Olindiidae). The freshwater jellyfish *C. sowerbii* is hypothesized to originate from the Yangtze River in China [20,21,22,23], and it has since spread to every continent except Antarctica [24,25,26,27,28]. In the United States, *C. sowerbii* has been reported in 44 out of 50 states across a range of freshwater ecosystems.

**Figure 1:**
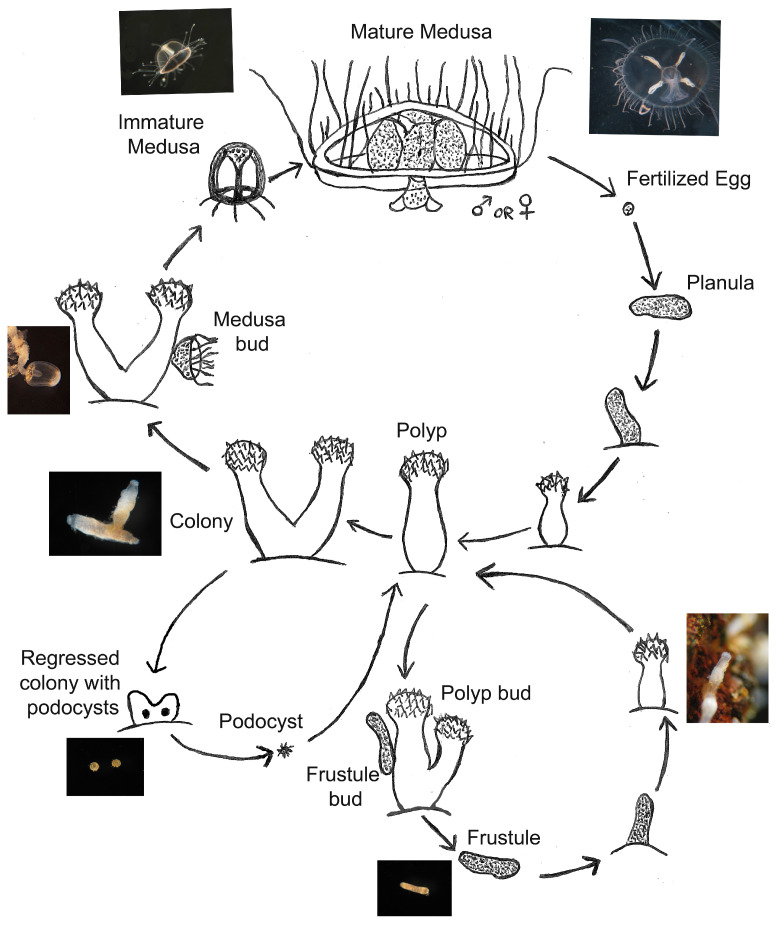
A modified life cycle from Folino-Rorem et al. [30] of the asexual, sexual, and dormancy stages of the invasive freshwater hydrozoan *Craspedacusta sowerbii*. Asexual reproduction occurs via a polyp budding new polyps (approx. 1 mm in height) or by producing a frustule (avg. length, 0.43 mm) that becomes a new polyp. Medusae (<1 mm) are also budded from polyps. Once sexually mature (approx. 15–20 mm), medusae spawn either eggs or sperms leading to a planula stage. In the dormant stage, a podocyst (avg. diameter 0.18 mm) occurs in unfavorable conditions. The sketch is not drawn to scale.

The life cycle of *C. sowerbii* consists of an asexual stage and a sexual stage [29,30] (Figure 1). Both portions start with the polyp stage: the most predominant form of *C. sowerbii* [31,32]; this stage includes both single-polyp animals and multiple-polyp colonies, both of which are referred to as “polyps” in this paper and in some of the literature [31,33]. In the asexual portion, polyps bud rod-like structures known as frustules, which then transform into a new polyp [34,35]. Polyps may also bud more polyps that remain attached to the original polyp, forming a colony. The sexual portion involves polyps budding motile medusae, which mature and produce gonads [19]; much like other cnidarians, these gonads combine to form a planula larva, which develops into a new polyp. These medusae are the larger stage of the life cycle of *C. sowerbii* and are most often the form identified when confirming the presence of *C. sowerbii* in a given body of water [31,33,36].

Sampling of *C. sowerbii* has mainly been performed on the medusa stage. This is for a variety of reasons; the medusae are larger and easier to see, so they can be easily acquired in several different ways, including scooping them directly out of the water with a jar or spoon [24,37,38,39,40], or towing a plankton net [18,26,40,41,42,43,44,45]. However, medusa sightings alone cannot confirm the establishment of *C. sowerbii* in a given environment [33]. Medusa appearances are sporadic [32] and can be influenced by a variety of environmental factors. We are aware of reports citing their appearance yearly at certain sites (Cumming R., Coal City, IL, pers. comm., 2022; Faith G., Belknap, IL, pers. comm., 2022) [46,47], while other sites can go many years without their appearance (Trostrud P., pers. comm., 2023) [48]. Additionally, Duggan and Eastwood [33] reported finding *C. sowerbii* polyps in bodies of water where the medusae had not been previously sighted, as well as finding no polyps in bodies where the medusae had been sighted. As such, Duggan and Eastwood [33] concluded that medusa sightings alone are not sufficient to establish the presence of *C. sowerbii* in a body of water, and they highlighted the importance of determining the presence of polyps. With rising temperatures, we might expect more appearances of the medusa stage [1], but medusa sightings cannot be guaranteed even if the polyp stage is confirmed to exist in a given environment. As such, reliable detection of the presence of *C. sowerbii* depends on confirmation of the polyp stage.

Polyp sampling poses several difficulties; for one, the polyps are sessile and must be sampled by pulling substrates out of the water. As such, current sampling methods mainly involve removing natural substrates (including rocks, plants, and wood) from the water and examining them under a dissecting microscope [24,33,49,50]. Polyps are also transparent and can be hard to detect, especially with untrained eyes [49]. Few studies have rigorously sampled to obtain *C. sowerbii* polyps, and far less work has been performed to collect and conduct experiments with the polyp and frustule stage of *C. sowerbii* compared to the medusa stage [33,35,51].

While eDNA is used to detect *C. sowerbii* [52,53], an eDNA-based methodology of detection may be more skill-intensive and could require more funding [54,55]; therefore, it may not be widely accessible. Additionally, recent studies utilizing eDNA samples in conjunction with hydrobiological surveys have detected *C. sowerbii* via eDNA surveys but have not located the morphological stages of frustules, polyps, or medusae [56,57] (Darling J., pers. comm., 2024) [58]. As such, the detection of the actual microscopic polyp stage remains challenging.

Due to the difficulty but importance of sampling the polyp stage of *C. sowerbii*, more insight into the efficacy of sampling is warranted. However, to our knowledge, there exists no systematic field method for collecting polyps other than transporting natural substrates to the laboratory for analysis using a microscope. As such, we sought to evaluate the capabilities of various sampling techniques to sample for *C. sowerbii* polyps by formulating a methodical and effective sampling method and providing quantitative data on sampling efficacy for use in future sampling and research. We deployed four substrate types (glass and plastic microscope slides, Hester-Dendy discs, and small glass Petri dishes) at three locations (three lakes in the Illinois–Indiana region, USA) to determine if these setups would promote polyp recruitment for collection purposes. Based on our prior experience with cultivating *C. sowerbii* on glass and plastic slides, we expected to find polyps on the glass and plastic slides and the glass Petri dishes. To the best of our knowledge, we are unaware of polyp collection on Hester-Dendy samplers.

## 2. Materials and Methods

Our work aims to address the previous difficulties involved in collecting the polyp stage of *Craspedacusta sowerbii*; thus, we constructed an apparatus and used a methodological protocol allowing for ease of deployment, retrieval, and subsequent analysis.

### 2.1. Pilot Laboratory Substrate Study

While various studies have described *C. sowerbii* polyps grown on glass [29] and plastic [59], we found no comparison between the two on its effects on the establishment and spread of the polyps. As such, we performed an experiment to assess the ability of culturing polyps on glass versus plastic slides in aquaria. We cultured frustules from Coal City, Illinois, USA on glass slides (n=14) and plastic slides (n=15) (all 7.6 cm × 2.5 cm); to do this, we seeded the slides with ten frustules each in dishes of Hydra Medium (HM) [30]. Once two or more frustules were transformed into polyps, we put the slides into slide boxes (14.1 cm × 9.2 cm × 3.6 cm) with the front and back walls cut out in a technique adapted from Folino-Rorem and Renken [60]. These boxes were then placed into 2.5 L tanks of HM with filtration by an AquaClear 20 filter and aeration by an air stone and bubbler, with one tank for plastic slides and another tank for glass slides. To feed the polyps, these boxes were transferred to interim containers of HM and flooded with brine shrimp rinsed in deionized water. Polyps were fed for 2 min before being returned to the original tanks. Polyps were grown on slides in the tanks for 2 months before being counted. Since the slides were seeded with frustules on one side, we only recorded polyp numbers and frustule numbers for the top side of the slides.

### 2.2. Construction of Sampling Apparatus

**Figure 2:**
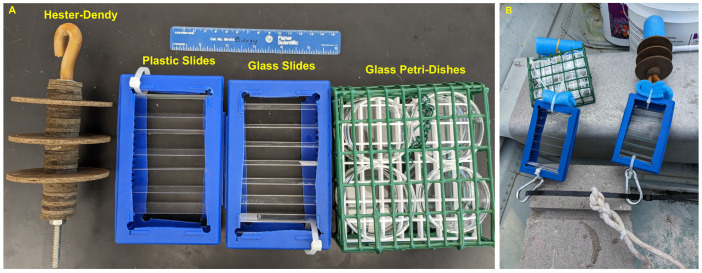
Construction of the sampling apparatus and substrate deployments used to collect *Craspedacusta sowerbii* polyps. (**A**) The substrates utilized in the apparatus, including a Hester-Dendy apparatus with three evenly spaced masonite discs separated by smaller masonite discs, microscope slide boxes with the sides cut out used to hold glass and plastic slides, and a metal cage holding eight Petri dishes (with four visible). (**B**) The entire setup with all substrates was attached to a zip tie looped through a half cinder block. All photos are by N. Folino-Rorem.

After observing high growth levels on both the glass and plastic slides in aquaria, we sought to address substrate types for field sampling. We chose four different substrates for sampling: glass slides, plastic slides, masonite Hester-Dendy discs (7.6 cm in diameter and evenly spaced apart, with discs of 2.5 cm in diameter), and borosilicate glass Petri dishes (6 cm diameter × 1.5 cm depth) in a metal cage (a bird suet cage, 13.5 cm × 12 cm × 5 cm). The use of glass Petri dishes is a technique used to sample for freshwater bryozoans and has been effective in collecting the frustules and polyps of *C. sowerbii* inadvertently (Wood T., pers. comm., 2024) [61]. These substrate types were selected based on our experience culturing *C. sowerbii* polyps in a laboratory setting and sampling techniques used for other aquatic invertebrates. In addition, we chose substrates that can be easily placed in a small dish of water for examination using a dissecting microscope. For our glass and plastic substrates, we used the aforementioned slide box apparatus (Figure 2A). Each slide box (14.1 cm × 9.2 cm × 3.6 cm) also had six glass microscope slides at the bottom of the box to help with weighing the boxes down; any organisms on these slides were not quantified.

To deploy these, we secured a zip tie through a cinder block that would rest on the bottom of the given pond or lake. We then attached one slide box of glass slides, one slide box of plastic slides, one Hester-Dendy sampler with three discs, and one metal cage with the Petri dishes to this cable with carabiners (Figure 2B). Blue pool noodle pieces were added to keep the items suspended while deployed. The slide boxes and Hester-Dendy discs were suspended vertically so the slides and discs were horizontal. The metal cages with the Petri dishes were attached in a way to keep the cages and, therefore, the dishes horizontal, and the openings of each dish faced outward in the cages. We also attached a rope to the zip tie to hold a buoy for the marking and collection of these setups later on (Figure 2B).

### 2.3. Field-Site Descriptions

**Figure 3:**
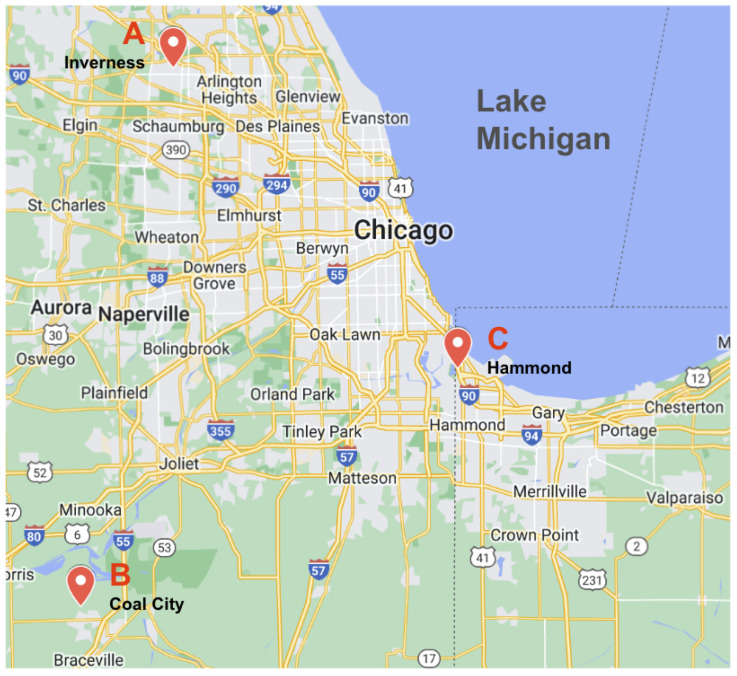
Approximate locations for the sampling sites, with major cities in the Northern Illinois–Indiana region labeled: (**A**) Inverness (IL), (**B**) Coal City (IL), and (**C**) Hammond (IN).

We sampled three locations in the Illinois–Indiana (USA) region (Figure 3, Table 1), two of which have confirmed both polyps and medusae present (Inverness and Coal City, IL, USA). Documented medusae sightings for Inverness were in 2012 (Trostrud P., pers. comm., 2024) [48] and in 2020 for Coal City (Cumming R., pers. comm., 2024) [46]. The third location for sightings was in Hammond, Indiana (IN), is Wolf Lake. To our knowledge, neither the polyp or medusa stage of *C. sowerbii* have been confirmed at Wolf Lake (Peard T., pers. comm. 2012) [62], so we were especially interested in seeing how our setups may document the presence of polyps at this location. These three locations are similar in water parameter values such as Secchi depth, conductivity, temperature, and the pH recorded for similar temperature locations of *C. sowerbii* [36].

The first location is a shallow drainage lake, Lake Harrowgate, in a neighborhood in Inverness, Illinois. The site has an input of cold water with high dissolved oxygen from the bottom of the lake. In 2022, we collected the polyp stage by scraping pieces of wood from submerged trees. The water is turbid with a Secchi depth of 0.75 m. The water conductivity is highest at this location compared to the other two locations, indicating more salts and minerals due to runoff from the surrounding homes (Table 1).

The second location is a large pond in a private club in Coal City, Illinois. The grounds consist of several lakes where *C. sowerbii* medusae are often sighted by fishermen, including the Coal City Area Club Catfish Pond where we sampled. We recently confirmed the presence of polyps in prior samples of wood and stems from this lake. The pond has a low turbidity with a Secchi depth of 2 m. In addition, the Coal City Area Club Catfish Pond is a calm body of water surrounded by tall grasses and is regularly used for human recreation, especially for fishing.

The third location is a public lake, Wolf Lake, which straddles the Indiana and Illinois state line. We deployed our setups on the Indiana side in Hammond, Indiana. The lake is regularly used for recreation (especially fishing) and has a Secchi depth reading of 1 m. Wolf Lake is larger than the other two locations.

### 2.4. Setup Deployments and Counting

We deployed four setups at each of the three locations. At each location, two different sites were identified, and two setups were placed at each site and were just off the lake or pond bottom by being attached to the cinder blocks. The sites were selected based on proximity to submerged natural substrates such as trees, rocks, and plants. At Inverness, previous sampling of *C. sowerbii* polyps occurred in 2022; therefore, we deployed the setups near submerged trees known to have polyps. The water depth was 2–3 m. At Coal City, we placed 2 setups closer to the shoreline near plants and twigs (Site 1) and placed 2 setups offshore in the middle near a rocky bottom (Site 2). In contrast, polyps had not been located at Hammond (Wolf Lake) prior to deploying our setups, so we selected spots where we hypothesized polyps to be on natural substrates. Setups were left at each location for two months before we retrieved them; while our setups at Coal City and Inverness were retrieved without difficulty, we lost two of the setups at Site 1 at the Hammond location due to vandalism, altering the total number of substrates analyzed (Table 2 and Table A1).

Once removed from the water, we removed the four substrates from the cinder block and placed them into separate containers filled with water from the site. These samples were then transported back to Wheaton College (Wheaton, IL, USA) for examination. We used a dissecting microscope to search all slides, Hester-Dendy discs, and Petri dishes for polyps and frustules; each side of each substrate was counted separately (so one slide, Hester-Dendy disc, or Petri dish yields n=2, and there was a total sample size of n=446 across all substrates at all sites). Additionally, any accumulated sediment on the slides was gently puffed away using a pipette. Once the number of polyps and frustules on each substrate was counted, we standardized their numbers so as to obtain a per cm${\;\;}^{2}$ count of the polyps and frustules for each substrate type.

We identified general types of other invertebrates observed on the various substrate types and identified 4 major categories, which we referred to as tubes (aquatic insect larvae and oligochaete tubes), *Hydra*, bryozoans, and other invertebrates (planaria, snails, and limpets). Additionally, we kept some microscope slides and Petri dishes with podocysts from all 3 locations in HM and fed them *Artemia* brine shrimp rinsed in deionized water [30] for continued monitoring and examination.

### 2.5. Follow-Up Laboratory Experiment

We performed a small follow-up experiment related to the sampling procedure. Firstly, while the ability of frustules to attach and develop into polyps was tested on plastic and glass, we had not confirmed it on Hester-Dendy discs; as such, we seeded the Inverness frustules on Hester-Dendy discs suspended in HM, and we monitored them for both attachment and development into polyps. We had 3 replicate bowls each with one disc that was seeded with 15 frustules from the Inverness population. This was conducted with older discs and with recently purchased discs to see if it was a factor in establishment.

### 2.6. Statistical Methods

All statistical analyses were conducted with RStudio version 2023.09.1+494.To assess the differences between the polyps and frustules that settled on glass and plastic slides in our preliminary laboratory studies, we first performed normality checks with Shapiro–Wilk tests, and we then performed further analysis with *t*-tests accordingly.

To assess the differences between polyps, frustules, and other invertebrates that settled on our four different field sampling substrates, we performed similar normality checks with Shapiro–Wilk tests. Following this, we performed Kruskal–Wallis and Dunn post hoc tests using R and the dunn.testpackage [63]. All differences were considered statistically significant with a *p*-value of <0.05 after Bonferroni correction.

## 3. Results

### 3.1. Pilot Laboratory Substrates

In general, we observed more polyps and frustule presence on the glass slides (Table 3). The experimental groups for the polyps per slide and frustules per slide followed normal distributions by Shapiro–Wilk tests (Table A2), so we used *t*-tests to compare the samples on glass slides versus on plastic slides. We found no significant difference in the number of polyps per slide (t=1.52,df=20.32,p=0.15) or in the number of frustules per slide (t=0.85,df=21.33,p=0.41).

### 3.2. Field Results

**Figure 4:**
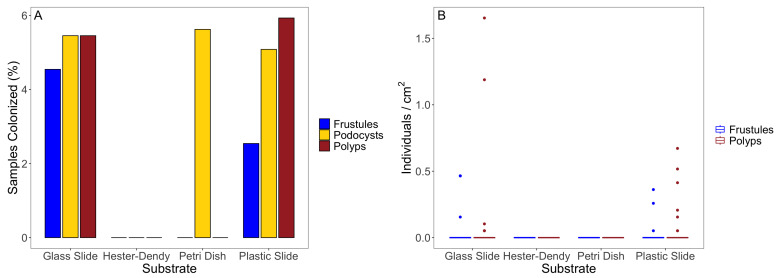
The colonization and densities of the *Craspedacusta sowerbii* stages on the four substrate types. (**A**) Percentage of the the sampled substrates found to have frustules, podocysts, or polyps. (**B**) Distributions of the polyps and frustules per cm${\;\;}^{2}$. Since a small percentage of samples were colonized, the boxplot display indicates the medians (horizontal lines) and respective outliers (full circles).

The field results indicate that our method is capable of recruiting the benthic frustule and polyp stages of *C. sowerbii*, and we found frustules and polyps on both glass and plastic slides, but we did not find them on the Hester-Dendy discs or on the Petri dishes. However, we generally had low polyp and frustule numbers overall, and a large portion of our deployed substrates remained uncolonized (Figure 4A); because of this, neither polyps per cm${\;\;}^{2}$ nor frustules per cm${\;\;}^{2}$ followed a normal distribution within any experimental group (Table A3). A Kruskal–Wallis test and Dunn post hoc test indicated a significant difference between the number of polyps per cm${\;\;}^{2}$ between all methods (H=12.94,p<0.001,df=3) (Table A4). Additionally, the plastic and glass slides had more polyps on them compared to the Hester-Dendy discs and Petri dishes (Figure 4B). Similar results were observed when quantifying the differences in the number of frustules per cm${\;\;}^{2}$ between substrates (H=9.20,p=0.03,df=3) (Figure 4B, Table A5).

We also compared the polyps recruited at the different locations. More specifically, while we found polyps at the Inverness and Hammond locations and frustules at the Inverness location, we did not find polyps or frustules at the Coal City location, but we did find podocysts on three slides and on one Petri dish at Coal City. There was a statistically significant difference in polyps per cm${\;\;}^{2}$ between the sites (H=16.74,p<0.001,df=2) (Table A6). Since we only found frustules at the Inverness location, we did not perform a comparison between the frustules at the different sites (Table A3).

**Figure 5:**
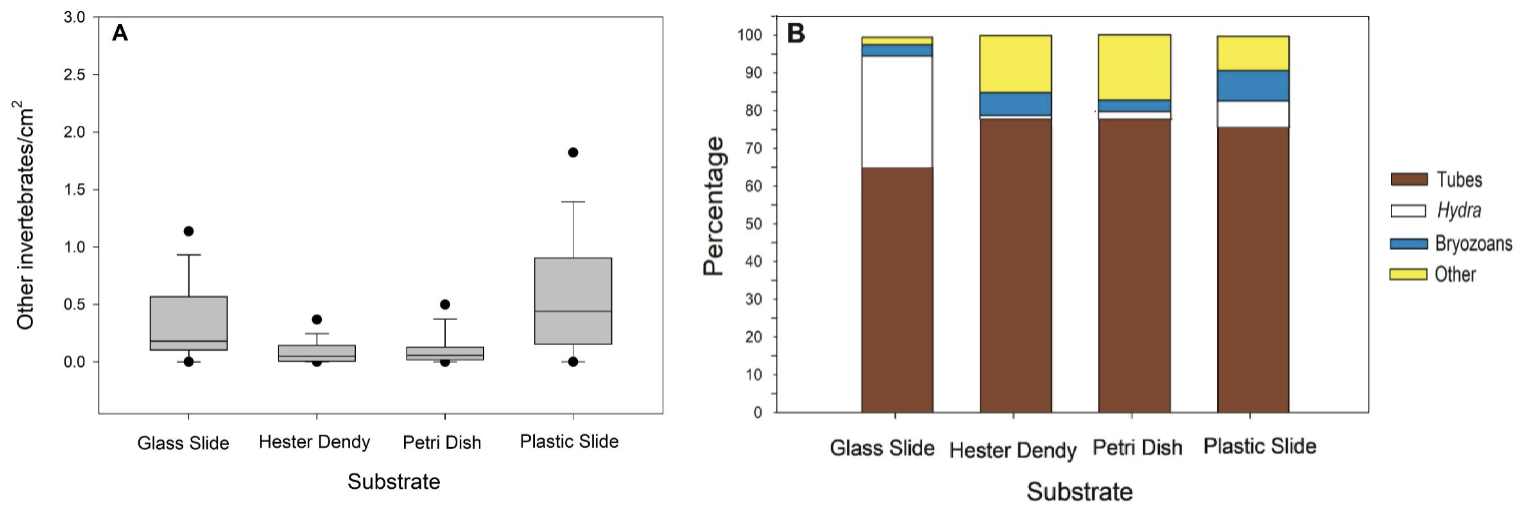
Graphicalrepresentations of the invertebrates (excluding *Craspedacusta sowerbii*) colonizing the different substrate types. The four major categories are referred to as tubes (aquatic insect larvae and oligochaete tubes), *Hydra*, bryozoans, and other invertebrates (planaria, snails, and limpets). (**A**) Boxplot display of the interquartile range of the data, with whiskers indicating the lowest and highest values. The horizontal line within a box represents the median and full circles specify outliers. All pairwise comparisons were significant at *p* < 0.05 (Kruskal–Wallis with Dunn post hoc test after Bonferroni correction) (Table A7). (**B**) Percentages of the representative invertebrates found on the substrates.

Furthermore, we were able to identify various types of invertebrates that had also settled on the slides, including oligochaetes, *Hydra*, bryozoan colonies, dreissenid mussels, and chironomids. The number of invertebrates per cm${\;\;}^{2}$ did not follow a normal distribution for any experimental group (Table A3), so a Kruskal–Wallis test indicated a significant difference in the number of other invertebrates per cm${\;\;}^{2}$ (H=137.22,p<0.001,df=3) (Table A7), with Hester-Dendy discs containing the highest number of invertebrates compared to all other groups (Figure 5A). In addition, the percentages of tubes (aquatic insect larvae and oligochaete tubes), *Hydra*, bryozoans, and other invertebrates (planaria, snails, and limpets) were determined for the four substrate types; clearly, the tube-building invertebrates were the predominant invertebrates. *Hydra* were present on both types of slides and less so on the Petri dishes and Hester-Dendy discs (Figure 5B). The tubes (aquatic insect larvae and oligochaete tubes) were most abundant on all four substrate types at Coal City.

Additionally, during the analyses of all substrate types, we found podocysts on the glass slides and Petri dishes from all three sites, as well as on the plastic slides from Wolf Lake (Figure 4A). While we did not collect any quantitative data on the number of podocysts per cm${\;\;}^{2}$, we found that some of the podocysts had regenerated into polyps after being cultured at room temperature (22 ${\;\;}^{\circ }$C).

### 3.3. Follow-Up Experiment Results

Follow-up experiments provided additional insights to some of our findings. When *C. sowerbii* frustules were seeded on Hester-Dendy discs, we found that the frustules failed to attach to the discs. Instead, the Hester-Dendy discs had tinted the water brown and the frustules disintegrated. This occurred with both the new and older discs.

## 4. Discussion

Our findings are indicative of the deployment of glass and plastic slides as an effective sampling method for detecting and collecting frustules, polyps, and even podocysts of *Craspedacusta sowerbii*. Given the challenges and time involve in finding them compared to the conspicuous medusa stage, these setups are promising for increasing the ability to definitively locate these life-cycle stages of *C. sowerbii* in various locations globally. Additionally, our results indicate that cultivating polyps on glass versus plastic may be more favorable for the establishment of polyps and frustules. While glass slides had more polyps and frustules settle on them in a laboratory setting, this difference was not statistically significant (Table 3).

This sampling apparatus can also be effective for obtaining specific populations of *C. sowerbii*, as well as other freshwater cnidarians that may be difficult to sample due to inconspicuous life-cycle stages, such as *Astrohydra* and *Limnocnida*, both with smaller polyps than *C. sowerbii*, and there is a need to determine more about possible frustule and podocyst stages for these two genera [64]. Perhaps the limited distributions of *Astrohydra* (Japan) and *Limnocnida* (Africa and India) are not as limited as we think, and our sampling setup could prove beneficial in collecting the life-cycle stages of *Astrohydra* and *Limnocnida* [64]. Furthermore, these setups could be utilized and be effective in collecting smaller invertebrates’ life-cycle stages in freshwater and marine systems, especially since planulae with cilia are free swimming and disperse much more easily compared to the frustule of *C. sowerbii*, which is tethered to the polyp via a mucous thread [34]. Marine settlement studies typically utilize PVC biofouling plates [65,66]. Furthermore, our sampling apparatus has the potential to locate and/or identify the presence of stages of cryptic non-indigenous species (NIS) such as bivalves, bryozoans, bivalves, and hydrozoans in marine and freshwater benthic habitats for early detection, and they are smaller in size and easier to analyze [67].

In both the laboratory and field aspects of this work, plastic slides were an effective substrate for the establishment of polyps. Increasingly, plastics are of great concern in aquatic habitats. The effects of ingested microplastics and how macroplastics serve as substrates for invertebrates have been given much more attention in marine ecosystems [68,69]. Researchers have assessed the selection for and colonization of plastics in the life-cycle stages of scyphozoans and other invertebrates such as bryozoa and tube-dwelling annelids [70,71,72,73]. The colonization or fouling of plastics by these invertebrates and the rafting or dispersal enhanced by plastics is also of concern in the spread of invasive species such as bryozoa and hydroids in marine systems [73,74]. Furthermore, there is increased attention toward plastics in freshwater systems since rivers often feed plastics into marine habitats [69,75,76,77]. Our sampling apparatus with plastic slides or modifications to include similar plastic substrates could enhance potential freshwater ecosystem research by addressing substrate type preferences for specific genera, effects of plastics (especially bottles) on invertebrate health, and the spread of invasive species.

Research addressing the location of the non-medusa stages of the life cycle of *C. sowerbii* has focused on locating the polyp stage [31,32,33]. The four substrates recorded in the literature are wood [24,33,49,50], stones [30,33,49,50], plant material [78,79,80], and dreissenid shells [34]. Collecting these substrates as a way to locate non-medusa stages has drawbacks by being labor and time intensive. Natural substrates are difficult to examine with a dissecting microscope due to their size, and it is difficult to detect the transparent polyp stage on natural substrates due to substrate opacity. Duggan and Eastwood [33] reported examining 10–15 collected stones four times over a two week period when looking for *C. sowerbii* polyps—they did not report seeing frustules and/or podocysts on the stones. Klotz [49] noted the challenge of collecting stones and searching for polyps that are small and transparent, and they suggested staining polyps to increase visibility. Dr. Wood (pers. comm., 2024) [61] easily located and identified these life-cycle stages of *C. sowerbii* while conducting bryozoa fieldwork, and they stated that frustules and polyps are much easier to locate on glass compared to natural substrates.

Compared to all these natural substrates, we found that our method mitigates many of the problems presented by collecting natural substrates such as accessing, transporting, and examining the wood, rocks/stones, and vegetation from a habitat. The use of microscope slides, small Petri dishes, and Hester-Dendy discs allows for quick and easy examinations for frustules and polyps since these substrates fit in dishes that are appropriate for use with a dissecting scope compared to larger natural substrates such as rocks and wood.

In addition to being small and transparent, polyps are often covered with algae and detritus, making them difficult to locate [34] (Wood T., pers. comm., 2024) [61]. Because of the substrate type and shape, we easily observed all three stages the first time (in accordance with our results), and we easily observed frustules, polyps, and podocysts on the glass and plastic slides during the first time when searching the substrates by simply modifying scope illumination angles and brightness.

Additionally, our results indicate that much less time is required to examine the slides, Petri dishes, and Hester-Dendy discs compared to natural substrates. Our prior attempts of locating polyps have been time consuming. Small logs from Catfish Pond in Coal City that were approximately 0.5 m in length were cut to fit into a cooler and returned to the lab. They were then cut into smaller portions approximately 8 cm in length to fit into a square dish and were then examined for frustules and polyps. Examining these small log portions took 2–3 h. Smaller stems and scrapes of wood from submerged logs were gathered at Harrowgate in Inverness and placed in coolers for transport. Two to four hours of examination using a dissecting scope lead to the location of a small patch of polyps (five polyps). We spent two days of 6 h/day examining several hundred dreissenid shells from Lake Michigan to locate two polyps and six frustules, which were often located in the byssal threads of the mussels. The examination of both sides of the five to six glass or plastic slides took approximately 25–30 min. Examining both sides of three Hester-Dendy discs took approximately 30–40 min, while the Petri dishes took the most time since there were a total of eight dishes and the top and bottom of each dish took 45–50 min to examine. Changing the lighting on a dissecting scope using fiber-optic lights enhances the ability to see these small and often transparent life-cycle stages of *C. sowerbii*. In addition to saving time, our setups permitted easier location/sittings of the frustules and polyps on glass substrates under a dissecting scope, and they also provided attached polyps for future laboratory culturing. Attached polyps on slides in cutout slide boxes can be placed in small aquaria and allow for the establishment of lab cultures for future experimental work on *C. sowerbii*.

Interestingly, we did not observe frustules or polyps on the glass Petri dishes as we might have expected (Wood T., pers. comm., 2024) [61]. Two reasons may explain this absence: One is that the cages with the Petri dishes were too high up in the water column from the half cinder block. The cages with the Petri dishes were positioned above the glass slides box (Figure 2B) and most likely not close enough to source substrates. The fact that the slide boxes were closer to the cinder block in all of the setups and closer to the benthic substrate where frustules were produced from source polyps may explain the minimal occurrences of frustules and polyps on the Petri dishes and Hester-Dendy setups. Future studies where all four substrates are alternated in position relative to the cinder block would address this issue. The second explanation, especially likely for the Coal City placement sites in the pond, is that the setups were not near or close enough to the natural substrates with established polyps. This can be rectified in future site location choices within a given aquatic habitat. We, therefore, suggest that field sampling utilize glass slides, though either glass or plastic will work, as well as glass Petri dishes (Wood T., pers. comm., 2024) [61].

Further improvements for this work would involve controlling the timing of apparatus deployment, i.e., when they are deployed and how long the apparatuses are left in the water. We were unable to retrieve the setups at different times in a given location and our setups were out for 2 months. The duration of 2 months for the Coal City apparatuses may have been too long, leading to the absence of frustules and polyps due to predation by chironomids, mites, and other invertebrates. We have often observed mites and chironomids eating polyps in laboratory cultures that contain wood gathered from a given location. Future research would entail leaving the apparatuses in the water for varying periods of times such as 2-week, 1-month, and 2-month periods to see if differences in frustule and polyp recruitment would be observed. Deploying setups for various lengths of time would provide a more complete documentation of frustule and polyp presence and production relative to water parameters such as temperature, pH, and turbidity. In conjunction, recording zooplankton and benthos food availability via bottom grabs along with abiotic parameters at a given location would provide valuable information for the food availability for various life-cycle stages.

We also suggest that the impact of additional abiotic and biotic factors can influence the spread and establishment of *C. sowerbii*. In the laboratory, we observed frustules floating in an aquarium and flowing toward the water intake tube of a side filter. Aquaria with various degrees of flow have been shown to be an effective method thorugh which to culture all stages of the life cycle [29] (pers. obs.). It is very conceivable that the flow in aquatic habitats can lead to frustule dispersal and may aid in the recruitment of frustules and polyps to new substrates away from the source polyps. Matthews [34] documented frustules spreading via thin mucus threads, and we have also observed this in the laboratory. Therefore, future studies subjecting polyps and frustules to specific flow velocities would address possible flow effects. Additionally, studies on marine cnidarians have focused on microbiotope variation due to depth differences, as well as on the orientation of polyps on substrate surfaces relative to currents/flow and the impact of the surrounding vegetation [36,70,81]. These ecological factors may be important for the settlement of *C. sowerbii* polyps in different types of aquatic habitats (e.g., rivers, lakes, and ponds) and warrant future investigation.

Furthermore, our results suggest that investigations into the nature of interactions between *C. sowerbii* and other organisms are also warranted for future study. More specifically, further research into the specific relationship between *C. sowerbii* polyps and dreissenids would be interesting. Stankovic and Ternjej [82] described *C. sowerbii* polyps on *Dreissenia polymorpha* shells, and we have often observed frustules and polyps on dreissenids in samples from Lake Michigan (pers. obs., N. Folino-Rorem). In this study, we found small mussels and polyps on the substrates deployed at Hammond (Wolf Lake) but no polyps on the mussels, likely due to the small size of the mussels. Dreissenids and other freshwater bivalves may provide additional substrate for polyps to become established, as seen with empty bivalve shells providing a means of locating polyps and podocysts with marine jellyfish (scyphzoans) [83]. However, with live mussels, additional factors could be at play, including flow created by dreissenid filtration and predation by chironomids and mites in mussel clumps. Further field and laboratory studies addressing these associations with bivalves would provide insights into the establishment dynamics of the benthic life-cycle stages of *C. sowerbii*.

The organisms we collected on our substrates include predators of *C. sowerbii* frustules and polyps, such as mites and chironomids, and other invertebrates that may compete with polyps for space such as bryozoans. Our Hester-Dendy disc samples featured higher numbers of other organisms such as oligochaetes, chironomids, and potentially other invertebrates compared to all other substrates. While it is conceivable that other organisms colonized the Hester-Dendy discs before the polyps and outcompeted them for space or preyed on frustules that recently settled, the Hester-Dendy discs we used could also have been less suitable for the settlement of frustules due to some degree of contamination (as stated in the Results Section 3.3).

Previous research addressing the use of new versus previously used Hester-Dendy discs have suggested that previously used discs yield more aquatic insects (EPT, Ephemeroptera, Plecoptera, and Trichoptera) compared to new discs [84]. The surface of older discs is less smooth than newer discs. As such, perhaps newer discs initially release a substance in the water that hinders the frustule or polyp establishment of *C. sowerbii*; thus, older discs could be more effective. Deploying both newer and older Hester-Dendy discs (prior use of 6 wks) closer to the cinder block near our known polyps sources would add insight in using Hester-Dendy setups for cnidarian recruitment. However, we still suggest that it is much easier to see and locate such small stages such as frustules and polyps on glass rather than a darker, non-transparent surface of tempered hardboard.

Furthermore, our sampling protocol could aid in the detection of the morphological life-cycle stages of *C. sowerbii* in combination with eDNA studies. Moore and Stewart [53] suggested that detecting *C. sowerbii* was difficult no matter which method was used, whether eDNA or acrylic settlement plates (15 cm × 15 cm), in the 10 lakes in the Hudson River watershed. They stated how time intensive the process was to visually inspect the settlement plates and in the documented hours that were designated to both field collection and eDNA work. Two other invertebrate-monitoring projects have obtained positive eDNA for *C. sowerbii* but have not obtained morphological evidence (Darling J., pers. comm., 2024) [58]. Our sampling apparatus is time saving, has smaller substrate type sizes, and is highly effective in locating the most challenging benthic life-cycle stages of *C. sowerbii*, i.e., the frustules, polyps, and even podocysts. Our method for detecting the pre-medusa stage can also enhance our understanding of which aspects of the polyp biology influence the medusa occurrences and biology [21,85]. This information would prove valuable in the overall invasion process because observing the medusa stage is unpredictable. This invasive species may ecologically impact aquatic systems as we consider the various factors important in potential jellyfish blooms with increasing water temperatures [1,23].

## 5. Conclusions

While we presented and tested an efficient methodology for detecting and collecting the frustule and polyp stages of the invasive freshwater hydrozoan *Craspedacusta sowerbii*, our method, nonetheless, can be applied and improved to enhance the study of the ecological dynamics of benthic invertebrate life-cycle stages. For improvements on this method, we wish to particularly mention controlling deployment timing and substrate positioning relative to polyp sources. Our method also had disadvantages such as potential vandalism to the deployed apparatuses and predation by other invertebrates consuming frustules and polyps, leaving only podocysts behind. Therefore, a combination of methods (such as sampling substrates in conjunction with eDNA) would be ideal for the detection and collection of the different benthic stages of *C. sowerbii*. Our results also open up key areas for further research regarding *C. sowerbii*, particularly regarding the influence of abiotic and biotic factors on the establishment and spread of the frustule and polyp stages of *C. sowerbii*. We also wish to mention a future research direction of the impact of plastics on the spread of *C. sowerbii* and aquatic invasive species as a whole.

Nonetheless, we hope to further utilize this method to detect and collect the polyp stage at more locations, and we believe that this tool can be instrumental in determining the full range of *C. sowerbii* and other jellyfish life-cycle stages (marine and freshwater) since medusa sightings alone cannot adequately clarify the presence of benthic life-cycle stages. Enhanced information regarding the presence of polyps and ability to survive and bud medusae will potentially clarify the sporadic observance of medusae at given locations and aid in understanding how benthic stages influence and explain potential blooms of marine and freshwater jellyfish. 

## Figures and Tables

**Table 1:** Water parameters, latitude, and longitude for each location. All parameters were recorded on the day of deployment. At each location, we deployed setups at two different sites.

Location	Setup Site	Latitude	Longitude	Depth (m)	Date of Deployment	Secchi Depth (m)	Conductivity (μS/cm)	Temperature	pH
Inverness (IL)	1	42.116540${\;\;}^{\circ }$ N	88.122080${\;\;}^{\circ }$ W	2	5 June 2023	0.75	1055.0	25.80 ${\;\;}^{\circ }$C	8.26
	2	42.116680${\;\;}^{\circ }$ N	88.122060${\;\;}^{\circ }$ W	3					
Coal City (IL)	1	41.316213${\;\;}^{\circ }$ N	88.271704${\;\;}^{\circ }$ W	3	22 June 2023	2	228.6	28.3 ${\;\;}^{\circ }$C	7.93
	2	41.316053${\;\;}^{\circ }$ N	88.271673${\;\;}^{\circ }$ W	4.5					
Hammond (IN)	1	41.671930${\;\;}^{\circ }$ N	87.511810${\;\;}^{\circ }$ W	3	18 July 2023	1	927.0	25.2 ${\;\;}^{\circ }$C	8.6
	2	41.672540${\;\;}^{\circ }$ N	87.512360${\;\;}^{\circ }$ W	3					

**Table 2:** The number of substrate types sampled at each location and the total number of each collectively. A more detailed breakdown of the substrate numbers deployed at each location can be found in Table A1. The lower replicate numbers for Hammond, IN, were due to the vandalism of the two setups deployed at Site 1.

Substrate	Coal City	Inverness	Hammond	Total
Glass Slides	24	19	12	55
Plastic Slides	24	23	12	59
Hester-Dendy Discs	12	12	6	30
Petri Dishes	32	32	16	80
Total	92	85	46	223

**Table 3:** Summary statistics of the number of polyps and frustules per slide for cultures of *Craspedacusta sowerbii* grown on glass slides versus plastic slides in the laboratory. All mean values per slide are ±1 SEM.

Substrate	Polyps	Frustules	Sample Size
Glass	28.29±5.98	24.71±20.12	n=14
Plastic	17.93±3.29	19.00±9.81	n=15

## Data Availability

All data gathered in this project, as well as the code used for subsequent analysis, can be found at the following GitHub repository: https://github.com/jonazhu/craspedacustasampling.

## Appendix A

**Table A1:** The experimental design providing the substrate types and replicates of each type deployed at each location. Each location had two different sites with two setups deployed at each site. Hammond originally had two different sites with setups deployed but the substrates at Site 1 were lost due to vandalism; these deployed substrates were not included in the total number of substrates sampled.

Location	Site	Setup	Glass Slides	Plastic Slides	Hester-Dendy Discs	Petri Dishes
Inverness	1	A	5	5	3	8
		B	4	6	3	8
	2	C	4	6	3	8
		D	6	6	3	8
Coal City	1	A	6	6	3	8
		B	6	6	3	8
	2	C	6	6	3	8
		D	6	6	3	8
Hammond	1 (Vandalized)	(A)	(6)	(6)	(3)	(8)
		(B)	(6)	(6)	(3)	(8)
	2	C	6	6	3	8
		D	6	6	3	8
Total Sampled			55	59	30	80

**Table A2:** Shapiro–Wilk normality test results for all the experimental groups in the preliminary substrate portion of this study (plastic slides and glass slides). Normality was tested on the two variables compared in this study, with those being *Craspedacusta sowerbii* polyps per slide and frustules per slide.

Substrate	Polyps per Slide	Frustules per Slide
Glass	W=0.920, p=0.218	W=0.905, p=0.132
Plastic	W=0.898, p=0.087	W=0.917, p=0.178

**Table A3:** Shapiro–Wilk normality test results for all the experimental groups analyzed in the sampling portion of this study (for the substrates of Hester-Dendy discs, Petri dishes, glass slides, and plastic slides; for the sites of Inverness, Coal City, and Hammond). Normality was tested on the three variables compared in this study, with those being *Craspedacusta sowerbii* polyps per cm${\;\;}^{2}$, frustules per cm${\;\;}^{2}$, and total invertebrates (aside from *C. sowerbii*) per cm${\;\;}^{2}$. N/A values indicate that all the values for that group were zero. Each side of each substrate was counted separately (so one slide, Hester-Dendy disc, or Petri dish yields n=2, and a total sample size of n=446 across all substrates at all sites); thus, the number of samples was twice the number of substrates deployed, as described in Table A1.

Feature	Group	Polyps/cm${\;\;}^{2}$	Frustules/cm${\;\;}^{2}$	Total Invertebrates/cm ${\;\;}^{2}$
Substrate	Hester-Dendy Discs (n=60)	N/A	N/A	W=0.957, p=0.034
	Petri Dishes (n=160)	N/A	N/A	W=0.724, p<0.001
	Glass Slides (n=108)	W=0.131, p<0.001	W=0.203, p<0.001	W=0.710, p<0.001
	Plastic Slides (n=118)	W=0.202, p<0.001	W=0.123,p<0.001	W=0.852, p<0.001
Location	Inverness (n=170)	W=0.172, p<0.001	W=0.197, p<0.001	W=0.826, p<0.001
	Coal City (n=184)	N/A	N/A	W=0.743, p<0.001
	Hammond (n=92)	W=0.080, p<0.001	N/A	W=0.793, p<0.001

**Table A4:** Results of the post hoc Dunn tests performed after Kruskal–Wallis tests comparing the number of *Craspedacusta sowerbii* polyps per cm${\;\;}^{2}$ on different sampling substrates. Bolded *p*-values were those results significant at p<0.05 after Bonferroni correction for multiple comparisons.

	Hester-Dendy Discs	Glass Slides	Plastic Slides
Glass Slides	Z=−2.019, p=0.022		
Plastic Slides	Z=−2.229, p=0.013	Z=−0.222, p=0.412	
Petri Dishes	Z=0.000, p=0.500	Z=2.617, p=0.004	Z=2.913, p=0.002

**Table A5:** Results of the post hoc Dunn tests performed after Kruskal–Wallis tests comparing the number of *Craspedacusta sowerbii* frustules per cm${\;\;}^{2}$ on different sampling substrates. Bolded *p*-values were those results that were significant at p<0.05 after Bonferroni correction for multiple comparisons.

	Hester-Dendy Discs	Glass Slides	Plastic Slides
Glass Slides	Z=−2.147, p=0.016		
Plastic Slides	Z=−1.199, p=0.115	Z=1.165, p=0.122	
Petri Dishes	Z=0.000, p=0.500	Z=2.782, p=0.003	Z=1.567, p=0.059

**Table A6:** Results of the post hoc Dunn tests performed after Kruskal–Wallis tests comparing the number of *Craspedacusta sowerbii* polyps per cm${\;\;}^{2}$ at different locations. Bolded *p*-values are those results that were significant at p<0.05 after Bonferroni correction for multiple comparisons.

	Inverness	Coal City
Coal City	Z=3.920, p<0.001	
Hammond	Z=2.727, p=0.003	Z=−0.497, p=0.309

**Table A7:** Results of the post hoc Dunn tests performed after Kruskal–Wallis tests comparing the number of invertebrates (besides *Craspedacusta sowerbii*) per cm${\;\;}^{2}$ on different sampling substrates. Bolded *p*-values are those results that were significant at p<0.05 after Bonferroni correction for multiple comparisons.

	Hester-Dendy Discs	Glass Slides	Plastic Slides
Glass Slides	Z=4.994, p<0.001		
Plastic Slides	Z=7.420, p<0.001	Z=2.829, p=0.002	
Petri Dishes	Z=11.109, p<0.001	Z=7.106, p<0.001	Z=4.163, p<0.001
